# Novel approaches to measuring knowledge among frontline health workers in India: Are phone surveys a reliable option?

**DOI:** 10.1371/journal.pone.0234241

**Published:** 2020-06-29

**Authors:** Neha Shah, Diwakar Mohan, Smisha Agarwal, Kerry Scott, Sara Chamberlain, Aarushi Bhatnagar, Alain Labrique, Meenal Indurkar, Rajani Ved, Amnesty LeFevre, on behalf of the Kilkari Impact Evaluation team

**Affiliations:** 1 Department of International Health, Johns Hopkins Bloomberg School of Public Health, Baltimore, Maryland, United States of America; 2 BBC Media Action, New Delhi, Delhi, India; 3 Oxford Policy Management, New Delhi, Delhi, India; 4 National Health Systems Resource Center, National Institute of Health & Family Welfare, New Delhi, Delhi, India; 5 Division of Public Health Medicine, School of Public Health and Family Medicine, University of Cape Town, Cape Town, South Africa; University of the Witwatersrand, SOUTH AFRICA

## Abstract

**Background:**

In 2017, India was home to nearly 20% of maternal and child deaths occurring globally. Accredited social health activists (ASHAs) act as the frontline for health services delivery in India, providing a range of reproductive, maternal, newborn, child health, and nutrition (RMNCH&N) services. Empirical evidence on ASHAs’ knowledge is limited, yet is a critical determinant of the quality of health services provided. We assessed the determinants of RMNCH&N knowledge among ASHAs and examined the reliability of alternative modalities of survey delivery, including face-to-face and caller attended telephone interviews (phone surveys) in 4 districts of Madhya Pradesh, India.

**Methods:**

We carried out face-to-face surveys among a random cross-sectional sample of ASHAs (n = 1,552), and administered a follow-up test-retest survey within 2 weeks of the initial survey to a subsample of ASHAs (n = 173). We interviewed a separate sub-sample of ASHAs 2 weeks of the face-to-face interview over the phone (n = 155). Analyses included bivariate analyses, multivariable linear regression, and prevalence and bias adjusted kappa analyses.

**Findings:**

The average ASHA knowledge score was 64% and ranged across sub-domains from 71% for essential newborn care, 71% for WASH/ diarrhea, 64% for infant feeding, 61% for family planning, and 60% for maternal health. Leading determinants of knowledge included geographic location, age <30 years of age, education, experience as an ASHA, completion of seven or more client visits weekly, phone ownership and use as a communication tool for work, as well as the ability to navigate interactive voice response prompts (a measure of digital literacy). Efforts to develop a phone survey tool for measuring knowledge suggest that findings on inter-rater and inter-modal reliability were similar. Reliability was higher for shorter, widely known questions, including those about timing of exclusive breastfeeding or number of tetanus shots during pregnancy. Questions with lower reliability included those on sensitive topics such as family planning; questions with multiple response options; or which were difficult for the enumerator to convey.

**Conclusions:**

Overall results highlight important gaps in the knowledge of ASHAs. Findings on the reliability of phone surveys led to the development of a tool, which can be widely used for the routine, low cost measurement of ASHA RMNCH&N knowledge in India.

## Background

### Introduction

In 2017, globally an estimated 18% of child deaths and 20% of maternal deaths occurred in India [1, 2]. Among Indian children under five, 60% of deaths occurred in the first 28 days of life and 50% were attributed to malnutrition [3, 4]. High rates of maternal and child mortality are underpinned by gaps in the quality and continuity of care received across the continuum of care from pregnancy to postpartum. In 2015–16, while 79% of women received at least one antenatal care visit, only 51% of women received the recommended four visits [3]. Similarly, while 81% of deliveries were conducted by a skilled provider, 35% of newborns did not receive a postnatal care visit by a skilled provider within two days of delivery [3].

Frontline Health Workers (FLHWs) are a vital strategy for improving access to timely and appropriate health information and health care in India and throughout low and middle-income countries (LMICs) where the majority of maternal and child deaths occur. In India, Accredited Social Health Activists (ASHAs) act as the bridge between the community and health system; serving as a health care facilitator, service provider, and health activist. ASHAs provide a range of health services including promoting universal immunization and providing referral and escort services for reproductive, maternal, newborn, and child health and nutrition (RMNCH&N) [5]. Since its launch in April 2005 as part of the National Rural Health Mission [6], the ASHA program has grown to include an estimated 938,000 ASHAs working across India’s 29 states; corresponding to an estimated two ASHAs per 3,000 population [7].

To qualify as an ASHA, a woman is expected to have at least 8^th^ standard education and complete an initial eight days of induction training, followed by another 20 days of skills-based training, provided in four rounds within the first 18 months of recruitment. The training includes content on basic RMNCH&N as well as nutrition and infectious diseases such as malaria and tuberculosis. This initial training is supplemented by 15 additional days of training annually, which includes refresher trainings and relevant new topics [8]. Following deployment, while ASHAs receive supervisory support, opportunities for in-service training vary sub-nationally, a factor which may have implications on the consistency of content and quality of the services ASHAs provide [9]. Indeed, one study found that ASHAs saw themselves as a cadre of healthcare services reporting to the medical supervisor rather than the Panchayat and community [10]. With the scope of ASHA activities expanding to include new health areas such as non-communicable diseases, new initiatives are needed to better evaluate the existing knowledge and competency levels of ASHAs. In response, strategies to address fundamental knowledge gaps can be implemented to improve service delivery.

Empirical evidence linking knowledge to service delivery and performance is emerging, but limited [11]. In India, assessments of ASHA knowledge have been small in scale and often constrained to a single topical area, including oral health [12], tuberculosis [13], and diarrhea [14]. Broader assessments of ASHA’s RMNCH&N knowledge and practices have pointed to significant gaps in knowledge about pre-eclampsia etiology [15], promotion of institutional delivery contraceptive-use [16], obstetric danger sign assessment [17], and neonatal care [18]. Collectively, this body of evidence reinforces the need to improve understanding of linkages between ASHA characteristics, knowledge, practices and service quality.

Efforts to measure knowledge amongst ASHAs have most commonly focused on structured surveys administered face-to-face, as one-off special surveys, or prior to / after receipt of training [19–24]. While these surveys offer important insights, they are often limited in frequency, and scale—largely in response to resource constraints. In the wake of near ubiquitous access to mobile phones amongst FLHWs, phone surveys are emerging as a potential low-cost alternative to face-to-face surveys. Some researchers have estimated that phone surveys may be less than half the cost of face-to-face alternatives [25]. Despite their potential, efforts to design phone surveys have varied [26, 27], and few analyses have been carried out to explore their reliability. The reliability analyses that are available have focused on response rates and associated implications for generalizability [28], rather than on the modality’s ability to capture the information precisely.

Drawing from surveys with ASHAs in four districts of Madhya Pradesh, India, this study aims to assess the determinants of RMNCH&N knowledge among ASHAs and examine the reliability of alternative modalities of survey delivery. We start by determining overall and domain-specific RMNCH&N knowledge scores for each ASHA and then seek to identify the characteristics of ASHAs and the health system associated with higher knowledge scores. We then assess differences in the reliability of the knowledge questions over different modalities including face-to-face surveys at two time points (test-retest) and caller attended telephone interviews (CATI; hereafter called phone surveys). Collectively, this body of work provides important insights into gaps in ASHA RMNCH&N knowledge, and most importantly, contributes to reliable face-to-face and phone survey tools, which can be used for the routine assessment of ASHAs.

## Methods

### Study setting

The study took place in four districts (Hoshangabad, Mandsaur, Rewa, and Rajgarh) of Madhya Pradesh (MP), a central landlocked state in India that is largely Hindi speaking, primarily Hindu, and mostly an agrarian economy [29]. Frontline health services are anchored by an estimated 75,000 ASHAs working across Madhya Pradesh’s 52 districts [30]. The study setting in MP is characterized by disparities in access to education—especially among women, literacy rates are lower in rural areas (urban: 78%; rural 51%); mobile phones owned by women (urban: 50%; rural 19%), and access to health services [31]. In 2015, only 35% of children were breastfed within one hour of birth and 58% of children exclusively breastfed until 6 months [31]; while one in four children under 5 experiencing wasting or thinness (weight-for-height), and 42% were stunted (height-for-age) [31].

### Study design and sampling

ASHAs in the selected study areas were randomly selected for participation in a cross-sectional face-to-face interview (n = 1,552). One ASHA per primary sampling unit, or village, was sampled as part of a larger impact evaluation of a mobile health program, Kilkari, targeting pregnant women in the same geographic area [32]. The sample size is sufficient to detect a 7% difference between any two groups in the overall knowledge score 50% or higher, assuming an alpha of 0.025, standard deviation of 0.18, and 0.80 power. In the parent evaluation, the sample size was calculated to detect a 7% difference in the overall knowledge score 50% or higher between the intervention and control groups.

A sub-sample of ASHAs interviewed during the cross-sectional face-to-face survey were re-interviewed 1–2 weeks following the initial survey to determine the degree to which repeated measurements in ASHAs interviewed (test-retest) provided similar answers. Reliability analyses of the face-to-face survey were used to streamline the survey tool to a length more manageable and focused on modules for which reliability testing was deemed necessary for implementation via the test retest. Assuming a *kappa* of 0.80, a margin of error of 0.05, an alpha of .05, and the proportion of positive responses of 0.35 for rater 1 and 0.40 for rater 2, 146 participants who have completed both surveys were required.

To develop a phone survey tool, ASHAs who had previously completed a face-to-face interview in the baseline survey 1–2 weeks prior were re-interviewed over the phone. The test-retest was deemed to be a reasonable length for a phone survey, so the same tool was used. The sample size requirements for the phone survey were the same as above: 146 completed interviews were needed.

### Data collection

The ASHA face-to-face survey included modules on demographic and work information, mobile phone ownership, use, and literacy, and experiences with Mobile Academy (a mobile health information training program). The questions were developed with the ASHA guidelines in mind as well the material available in training programs, such as Mobile Academy; questions were adjusted based on pretesting. The test-retest ASHA survey tool was a shorter tool as compared to the baseline face-to-face tool, but includes a subset of the same questions. The phone surveys were conducted with the same tool and methods to develop this are detailed elsewhere [33]. In brief, we assumed a step-wise approach starting with an expert driven approach to item generation, followed several iterations of piloting and translation before ultimately developing a large-scale face-to-face survey. To assess inter-rater reliability, we then repeated an abbreviated version of the same face-to-face survey amongst a sub-sample of respondents and administered the same tool via a CATI survey to a separate sample of ASHAs originally interviewed during the face-to-face survey. The main survey consisted of 10 male enumerators and 7 training days, and lasted from June to November 2018. The phone survey lasted 9 days with 1 day of training and two days of pilot testing. Three male enumerators conducted the phone survey. Both in person and phone interviews were conducted with the aid of the survey on tablets programmed using census Pro. The surveys included single response as well as multi-response questions. The multiple response questions were asked without prompting any response options but probing for other answers, and then selecting all responses mentioned on the tablet.

### Data analysis

**Fig 1:**
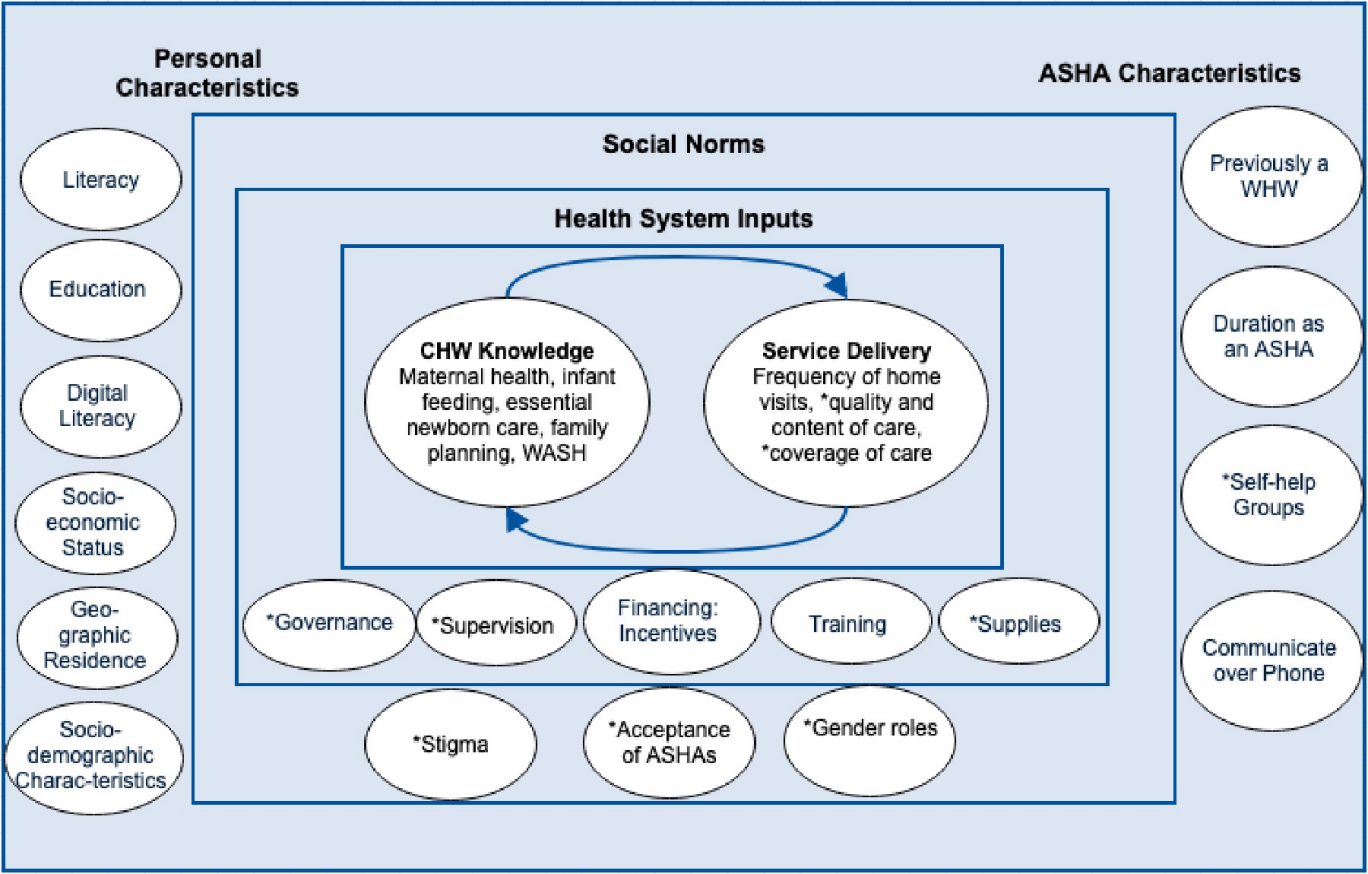
Conceptual Framework of determinants relating to knowledge of ASHAs.

All data were analyzed using Stata 15 [34]. Analysis of the determinants of ASHA knowledge was conducted through a multi-step process. Fig 1 presents a conceptual framework used to guide the analysis; it theorizes the relationship between personal characteristics, ASHA work-related characteristics, social norms, health system inputs, knowledge among ASHAs, and service delivery. This framework was adapted from the logic model generated by Naimoli et al [35] and further modified to include additional areas, such as social norms as described by Kok et al [36]. Those domains or topics with asterisks are ones for which we do not have data in our survey.

To assess determinants of ASHA knowledge, composite knowledge scores were created from 35 questions that were split into five separate domains: maternal health, infant feeding, essential newborn care (ENC), family planning, and WASH/diarrhea (Table 1). Within each domain, questions were given equal weight, and coded as 1 or 0 if there was one clear answer. If there were multiple correct options, each option was equally weighted so that if all correct options were picked, the score for that question would be equivalent to 1, but if 2 out of 3 correct options were selected, the score would be 0.66 for that question. The total score was calculated by summing all the individual domain scores. The scores were based on a scale of 0 to 100. Bivariate and multivariable analyses were conducted with the total score as the outcome variable. Independent variables were selected based on our conceptual framework (Fig 1). Multivariable analyses only included those variables that had an association with total knowledge score at a significance level of 0.20 or below during the bivariate analysis in an effort to avoid over-fitting the model [37]. β Coefficients from the adjusted regression model are presented with 95% confidence intervals.

**Table 1:** Questions included in each domain of knowledge.

Questions	Maternal Health	Essential Newborn Care	Infant Feeding	Family Planning	WASH diarrhea
1: What foods should a pregnant woman eat during pregnancy?	X				
2: How many tetanus injections should a woman have during pregnancy?	X				
3: How many IFA tablets should a woman take during pregnancy?	X				
4: Why should pregnant women take IFA tablets during pregnancy?	X				
5: What are some steps a pregnant woman and her family must take to prepare for delivery?	X				
6: What are some danger signs during pregnant for which pregnant women should go to a health facility immediately?	X				
7: How many times should you visit a new mother and her baby within one week of delivery?		X			
8: How many times should you visit a new mother and her baby within six weeks of delivery?		X			
9: When should the mother start breastfeeding after delivery?			X		
10: Why should she start breastfeeding them?			X		
11: How many times per day should newborn babies be breastfed?			X		
12: For how many months should baby be given only mother’s milk (not even water)?			X		
13: When should a baby be bathed for the first time?		X			
14: How should a baby be kept warm immediately after delivery?		X			
15: How soon after delivery should baby’s umbilical cord be cut? *Not included in scores due to differing and changed guidelines		X			
16: What should be used to cut the umbilical cord?		X			
17: What should be put on the cord after delivery?		X			
18: What are some important steps mothers must take to care for a preterm/LBW baby?		X			
19: What are some of the signs for which a newly delivered mother should seek care or go to a health facility?	X				
20: On average, how long should new mothers wait before they have another child?				X	
21: What are the benefits of using family planning?				X	
22: What are the modern family planning methods you know of?				X	
23: Right after delivery, before a new mother leaves the health facility, what family planning options are available to her and her partner/husband?				X	
24a: Male sterilization is an easy way to control family size				X	
24b: Men become physically weak after accepting male sterilization				X	
24c: PPIUCD insertion and female sterilization services can be free of cost at government facilities				X	
24d: PPIUCD has many harmful side effects				X	
24e: Women may never be able to have another baby if they use IUD for family planning				X	
24f: IUDs can be used for up to 10 years, if needed				X	
25: What is some information you share with women and injectable contraceptives?				X	
26: What is some information you share with new mothers about using oral contraceptive pills				X	
27: What should you give to a child to treat diarrhea?					X
28: When should a baby be given foods other than mother’s milk for the first time			X		
29: What type of food or liquid, other than breast milk, should a baby over six months be given?			X		
30: What are three critical times for a woman to wash her hands?					X
Total number of questions per domain	7	8	6	12	2

Reliability analyses were conducted with the unit of analysis being the individual ASHA. Kappa statistics were calculated to determine agreement between the two modalities tested in the test-retest survey and the phone survey. A kappa at or above 0.7 was considered to indicate moderate to strong agreement beyond chance [38]. To adjust kappa coefficients for differences in the prevalence levels of an indicator, as well as random and/or systematic differences between the two survey ratings, Prevalence Adjusted Bias Adjusted Kappa (PABAK) scores were calculated and are presented in the results. Prevalence indices account for differences in the prevalence of an indicator; where the prevalence is high, chance agreement may also be high and correspondingly, the kappa reduced [39]. PABAK scores between the face-to-face survey and the test-retest survey as well as PABAK scores between the face-to-face survey and the phone survey for each question gives us a sense of (a) the overall reliability of the question and (b) reliability of the question through the phone modality. A question was deemed reliable over the phone modality if the 0.7 kappa statistic threshold was met in both surveys.

### Ethics approval

Ethical approval for research activities in India was obtained from Johns Hopkins School of Public Health’s Institutional Review Board in Baltimore Maryland, USA and from Sigma Research and Consulting in New Delhi, India. All participants provided verbal consent before engaging in interviews.

## Results

### ASHA characteristics

ASHAs included in the cross-sectional face-to-face survey were a median of 35 years of age, 43% had three or more children, 76% were born in the district in which they currently work, 82% had completed 8^th^ standard or higher, and 82% could read a whole sentence (Table 2). Phone ownership was high: 90% owned a mobile phone and 12% owned a smartphone. A reported 40% of ASHAs had a government provided phone, while 68% had a government provided SIM. Among digital literacy indicators examined, 77% of the ASHAs were able to give a missed call, 73% store some or all contacts on a phone, 88% comprehended an IVR navigational prompt, 54% were able to navigate a phone to open an SMS, while 47% could both open and read an SMS. When asked about history of employment, 6% of ASHAs reported having previously worked as village health workers, and 60% had worked as ASHAs for 4–10 years. Awareness and receipt of mobile in-service training via Mobile Academy was low: 14% had heard of Mobile Academy, 11% had started Mobile Academy, and 10% had completed Mobile Academy. (Roll out of Mobile Academy in Madhya Pradesh has been poor as compared to other states, where according to analysis of system generated data, as much as 70% of ASHAs have completed Mobile Academy in states such as Rajasthan, Haryana and Himachal Pradesh.)

**Table 2:** Characteristics of ASHAs drawn from a face-to-face survey in 4 districts of Madhya Pradesh.

**Domain**	**Characteristic**		**Face-to-face Survey N (%)**
**Personal characteristics**	Sample Size		1,552
	Geographic District	Rewa	551 (35.5)
		Rajgarh	490 (31.5)
		Mandsaur	287 (18.5)
		Hoshangabad	224 (18.5)
	Caste	General	447 (28.8)
		Backward	744 (48.0)
		Scheduled caste or tribe	361 (23.3)
	Age (median (range))		35 (20–59)
	Married		1,455 (93.8)
	Parity	<3	863 (55.6)
		≥3	689 (44.4)
	Born in District		1,186(76.4)
	Highest level of Education	Completed ≤5^th^	275 (17.7)
		Completed 8^th^-10th	446(28.7)
		Completed ≥12^th^	831 (53.5)
	Literacy: Can read whole sentence		1,265(81.5)
	Socio-economic status	First wealth quintile	283 (18.2)
		Second wealth quintile	291 (18.8)
		Third wealth quintile	310 (20.0)
		Fourth wealth quintile	328 (21.2)
		Fifth wealth quintile	340 (21.9)
	Phone ownership and use	Own Mobile Phone	1,394(89.8)
		Own smartphone	193 (12.4)
		Daily phone use	1,483 (95.6)
		Can dial missed Call	1,190 (76.7)
		Stores contacts on phone	1,138 (73.3)
		Understood a recorded message	1,359 (87.6)
		Opened an SMS	838 (54.0)
		Read an SMS	728 (46.9)
**ASHA characteristics**	Previously worked as VHW		92 (5.9)
	Duration as ASHA	0–3 years	211(13.6)
		4–10 years	927(59.7)
		10+ years	414 (26.7)
	Communicate work over phone (mean score of 10)		0.39
**Health System Inputs**	Government provided Phone		623 (40.1)
	Government provided SIM		1,048 (67.5)
	Mobile Academy	Aware of it	217(14.0)
		Initiated	167 (10.8)
		Completed	158 (10.2)
**Service Delivery**	7 or more client visits a week		1,035 (66.7)

### RMNCH&N determinants of knowledge

**Fig 2:**
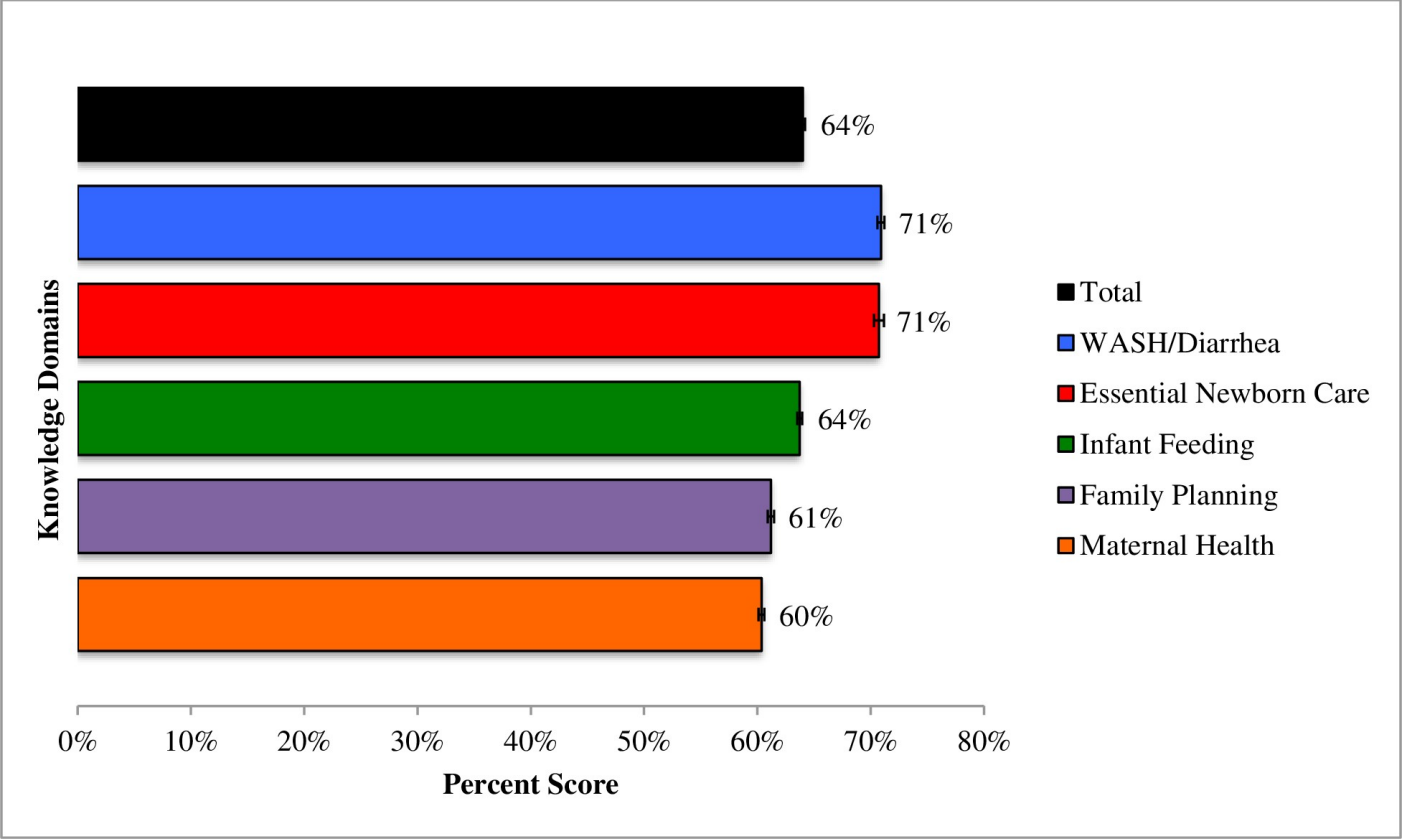
Mean ASHA scores for total overall knowledge and five RMNCH&N domains.

Fig 2 presents knowledge scores overall (64%) and across 5 domains: WASH/diarrhea (71%), essential newborn care (71%), infant feeding (64%), family planning (61%), and maternal health (60%). Simple and multivariable linear regressions were run to explore linkages between ASHA characteristics and mean RMNCH&N knowledge (Table 3). In the bivariate analyses, the majority of the characteristics were significant at the .2 significance level; the only exceptions were socioeconomic status, previous occupation as a village health worker, and parity, which were not significantly associated with ASHA knowledge. Findings from the multivariable model suggest that knowledge is significantly higher among ASHAs based in Hoshangabad (a district with greater proximity to urban areas), educated up to or beyond 12^th^ standard, below the age of 30, who have worked as an ASHA for a greater duration, own a phone, demonstrate comprehension of an IVR navigational prompt, and use mobile phones to communicate for work. Determinants of ENC knowledge (S1 Table) mirror these findings somewhat: ENC knowledge was significantly higher among ASHAs based in Hoshangabad, educated up to 12^th^ standard, and able to dial a missed call.

**Table 3:** Factors associated with reproductive, maternal, newborn and child health knowledge in 4 districts of MP, India.

Domain	Characteristics	Unadjusted coefficient (CI)	p-value	Adjusted Coefficient (CI)	p-value
**Personal Characteristics**	*District*				
	Hoshangabad	1.00	--	1.00	--
	Mandsaur	-2.35 (-4.96–0.26)	0.07	-1.69 (-3.64–0.26)	0.08
	Rewa	-2.58 (-5.40–0.25)	0.07	-2.30 (-5.05–0.46)	0.09
	Rajgarh	-7.78 (-10.54–5.03)	<0.01	-5.03 (-7.27–2.79)	<0.01
	*Caste*				
	General caste	1.00	--	1.00	--
	Other backward class	-1.25 (-3.43–0.94)	0.2	0.40 (-0.46–1.27)	0.33
	Scheduled caste or tribes	-0.52 (-3.15–2.10)	0.7	-0.31 (-2.03–1.40)	0.70
	Married	-1.55 (-3.22–0.13)	0.07	0.13 (-0.59–0.85)	0.70
	*Education*				
	Up to 5th standard	1.00	--	1.00	--
	Up to 8th standard	3.71 (-0.07–7.48)	0.054	2.43 (0.51–4.35)	0.02
	Up to 12th standard	5.38 (0.98–9.78)	0.02	3.58 (1.63–5.53)	<0.01
	Beyond 12th standard	10.61 (4.50–16.71)	<0.01	7.33 (3.31–11.34)	<0.01
	Number of children	0.04 (-0.34–0.42)	0.8		
	*Age*				
	< 30 years	0.69 (0.42–0.96)	<0.01	0.36 (0.13–0.60)	<0.01
	≥ 30 years	0.06 (-0.09–0.20)	0.4	0.01 (-0.09–0.11)	0.79
	Born in the district	-1.04 (-2.53–0.45)	0.2	-0.47 (-1.16–0.23)	0.17
	*Socioeconomic Status*				
	First wealth quintile	1.00	--		
	Second wealth quintile	1.46 (-1.17–4.08)	0.3		
	Third wealth quintile	1.47 (-1.05–3.99)	0.2		
	Fourth wealth quintile	0.86 (-2.33–4.05)	0.6		
	Fifth wealth quintile	2.01 (-1.19–5.22)	0.2		
	*Phone ownership and use*				
	Own phone	7.13 (4.21–10.05)	<0.01	2.80 (1.47–4.12)	<0.01
	Use phone daily	6.74 (0.89–12.59)	0.03	0.56 (-2.65–3.76)	0.71
	Own a smartphone	1.23 (-0.18–2.65)	0.08	-0.29 (-1.60–1.03)	0.65
	Able to dial a missed call	3.94 (1.00–6.89)	0.01	0.47 (-0.49–1.43)	0.31
	Able to understand an IVR navigational prompt	5.08 (2.02–8.14)	<0.01	2.79 (1.50–4.08)	<0.01
	Able to open and read an SMS	4.27 (2.08–6.46)	<0.01	1.16 (0.28–2.04)	0.01
	Store some or all contacts on phone	1.28 (-0.46–3.03)	0.14	-0.56 (-1.47–0.35)	0.21
	Use phone to communicate for work	23.01 (12.45–33.58)	<0.01	13.45 (7.89–19.01)	<0.01
**ASHA characteristics**	*Experience as an ASHA*				
	0–4 years	1.00	--	1.00	--
	5–10 years	2.38 (1.13–3.64)	<0.01	1.42 (-0.30–3.13)	0.10
	11+ years	2.90 (1.07–4.72)	<0.01	2.05 (0.48–3.63)	0.01
	Previously a VHW	1.28 (-1.31–3.87)	0.3		
**Health System Inputs**	*In-service training* Heard of Mobile Academy	5.61 (3.07–8.15)	<0.01	1.51 (-0.78–3.81)	0.18
	Started Mobile Academy	5.76 (3.35–8.18)	<0.01	0.29 (-5.22–5.81)	0.91
	Completed Mobile Academy	5.61 (3.10–8.12)	<0.01	-0.89 (-6.60–4.81)	0.74
	Government provided SIM	2.63 (0.91–4.36)	<0.01	0.37 (-0.82–1.56)	0.52
	Government provided phone	2.54 (0.51–4.56)	0.02	0.45 (-0.89–1.79)	0.48
**Service Delivery**	7 or more clients visits a week	2.38 (1.49–3.27)	<0.01	1.04 (0.38–1.69)	<0.01

### Survey reliability

Findings on the inter-rater reliability (test-retest) and intermodal reliability (face-to-face versus phone) are presented in Table 4 for reproductive and maternal health. Questions are displayed in the order in which they appear in the questionnaire; options are ordered from highest reliability (> 0.7 for both modalities) to only reliable in-person, and then unreliable options. Findings suggest that questions with the lowest reliability over both modalities were those pertaining to care-seeking for new mothers (k = .48), benefits of family planning (k = .54), impact of male sterilization (k = .64), and the harmful effects of and use of IUDs (k = .29). Questions with higher reliability in-person versus over the phone include those about number of IFA tablets taken during pregnancy (k = .71), difficulty breastfeeding being a danger sign for new mothers (k = .75), female sterilization (k = .76), and the rhythm methods (k = .75). Questions with the highest reliability face-to-face and over the phone included those about number of tetanus shots during pregnancy (k = .97), birth spacing (k = .85), and free IUDs at government facilities (k = .96). All kappa statistics reported here are the highest values of the two modalities for each option.

**Table 4:** Kappa statistic comparisons between the face-to-face survey and test-retest reproductive and maternal health knowledge questions.

Question	Options	Baseline Prevalence	Baseline prevalence among retest	Baseline prevalence among phone survey	Retest Pre-valence	Phone Survey Pre- valence	Retest Survey Adjusted Kappa	Phone Survey Adjusted Kappa
**Domain: Maternal Health**								
Food during pregnancy	Green Vegetables	0.99	0.98	1.00	0.97	0.99	0.93	0.99
	Fruits	0.90	0.86	0.95	0.94	0.86	0.73	0.74
	Dairy products	0.86	0.71	0.94	0.84	0.92	0.54	0.72
	Eggs	0.63	0.49	0.73	0.57	0.57	0.48	0.32
	Fish	0.50	0.45	0.62	0.55	0.48	0.47	0.29
	Meat	0.40	0.36	0.54	0.44	0.48	0.46	0.29
	Pluses and nuts	0.83	0.77	0.83	0.84	0.77	0.43	0.42
# of tetanus shots during pregnancy	2	0.96	0.90	0.97	0.94	0.98	0.85	0.97
# of IFA tablets during pregnancy	100	0.20	0.21	0.26	0.21	0.19	0.71	0.59
Reason for IFA tablets	Prevent/treat anemia	0.99	0.97	0.99	0.98	1.00	0.91	0.99
Reason for IFA tablets	Improve health of baby	0.88	0.87	0.88	0.88	0.63	0.63	0.14
Delivery Preparations	Clothes ready	0.94	0.91	0.97	0.96	0.95	0.82	0.86
	Identify transportation	0.83	0.82	0.89	0.93	0.90	0.60	0.65
	Emergency fund	0.80	0.68	0.81	0.82	0.58	0.51	0.26
	Identify place of delivery	0.26	0.13	0.23	0.25	0.19	0.49	0.43
	Register pregnancy	0.47	0.38	0.52	0.50	0.48	0.24	0.07
	Identify accompanying family members	0.28	0.28	0.35	0.51	0.11	0.10	0.33
	Have ASHA’s number	0.45	0.35	0.60	0.55	0.37	0.03	0.11
Cause for immediate attention for recently delivered women	Swelling limbs & face	0.80	0.73	0.79	0.77	0.85	0.48	0.45
	Vaginal discharge	0.41	0.30	0.52	0.35	0.23	0.47	0.17
	Decreased/absent fetal movements	0.25	0.31	0.30	0.31	0.08	0.43	0.45
	Dizziness & headache	0.64	0.64	0.66	0.73	0.75	0.39	0.23
	Vaginal Bleeding	0.72	0.64	0.81	0.76	0.62	0.36	0.30
	Jaundice	0.38	0.29	0.31	0.33	0.37	0.26	0.14
	Fever	0.41	0.36	0.45	0.53	0.67	0.26	0.02
	Convulsions	0.38	0.21	0.43	0.37	0.38	0.24	0.02
	Stomach cramps	0.55	0.54	0.64	0.57	0.36	0.14	0.10
Danger signs for new mothers	Scanty urine	0.05	0.02	0.06	0.08	0.00	0.80	0.87
	Placenta delayed delivery	0.08	0.04	0.08	0.06	0.00	0.85	0.83
	Difficulty breastfeeding	0.16	0.13	0.23	0.10	0.06	0.75	0.57
	Foul smelling vaginal discharge	0.17	0.09	0.15	0.13	0.09	0.60	0.64
	Depression, Psychosis	0.23	0.21	0.26	0.13	0.02	0.49	0.46
	Difficulty breathing	0.36	0.38	0.41	0.23	0.13	0.24	0.17
	Headache/blurred vision	0.65	0.56	0.67	0.62	0.67	0.19	0.20
	Convulsions/fits	0.53	0.48	0.55	0.51	0.55	0.17	0.03
	Severe abdominal/genital pain	0.41	0.40	0.51	0.59	0.23	0.17	0.14
	Loss of consciousness	0.44	0.35	0.38	0.41	0.45	0.14	0.05
	Fever	0.52	0.55	0.50	0.66	0.74	0.12	0.20
	Excessive vaginal bleeding	0.51	0.45	0.57	0.80	0.70	0.03	0.19
**Domain: Family Planning**								
Birth spacing	Wait for = > 3 years	0.92	0.84	0.94	0.89	0.96	0.76	0.85
Benefits	Time for current child care	0.91	0.87	0.94	0.84	0.65	0.54	0.33
	Easy way to control family size	0.74	0.69	0.75	0.79	0.46	0.43	0.02
	Financial savings	0.29	0.18	0.26	0.20	0.06	0.38	0.50
Modern methods	Oral contraceptive pills	0.97	0.97	0.99	0.98	0.98	0.91	0.95
	Lactational amenorrhoea method	0.02	0.02	0.04	0.01	0.00	0.93	0.92
	IUD	0.98	0.92	1.00	0.95	0.96	0.87	0.92
	Condom/Nirodh	0.98	0.94	0.98	0.97	0.94	0.85	0.90
	Withdrawal	0.07	0.04	0.06	0.05	0.00	0.86	0.88
	Female sterilization	0.92	0.91	0.95	0.91	0.81	0.76	0.56
	Rhythm method	0.20	0.08	0.25	0.13	0.00	0.75	0.51
	Injectables	0.81	0.65	0.85	0.83	0.78	0.41	0.46
	Male sterilization	0.84	0.69	0.92	0.77	0.83	0.29	0.57
Family Planning right after delivery	Withdrawal	0.00	0.00	0.01	0.01	0.00	0.99	0.99
	Rhythm method	0.01	0.00	0.02	0.02	0.00	0.95	0.96
	IUD	0.96	0.87	0.99	0.92	0.99	0.76	0.96
	Male sterilization	0.09	0.03	0.15	0.32	0.39	0.35	0.23
	Injectables	0.18	0.22	0.14	0.43	0.43	0.16	0.14
	Oral contraceptive pills	0.15	0.29	0.11	0.42	0.48	0.12	0.11
	Female sterilization	0.31	0.33	0.42	0.69	0.45	0.05	0.03
	Condom/Nirodh	0.25	0.39	0.23	0.54	0.46	0.03	0.14
Male sterilization controls family size	TRUE	0.97	0.94	0.98	0.92	0.97	0.77	0.91
Men become physically weak after male sterilization	FALSE	0.30	0.49	0.19	0.45	0.11	0.53	0.64
IUD & female sterilization free at gov facilities	TRUE	0.99	0.98	0.99	0.98	0.99	0.93	0.96
PPIUCD has many harmful side effects	FALSE	0.57	0.72	0.55	0.70	0.63	0.26	0.29
Women become sterile if they use IUD	FALSE	0.66	0.75	0.67	0.57	0.78	0.11	0.29
IUDs can be used for up to 10 years	TRUE	0.86	0.79	0.89	0.82	0.94	0.51	0.75
Information about injectables	Start 6 weeks after childbirth	0.08	0.03	0.04	0.05	0.00	0.84	0.92
	Safe for breastfeeding mothers	0.07	0.09	0.03	0.10	0.05	0.66	0.86
	Should be taken every 3 month	0.53	0.33	0.47	0.45	0.43	0.39	0.24
	Protection from pregnancy for 3 months	0.47	0.39	0.35	0.29	0.32	0.32	0.32
Information about oral contraceptives	Mala-N/OCP should be taken as prescribed	0.93	0.92	0.98	0.92	0.94	0.73	0.85
	Safe options for breastfeeding mothers	0.15	0.07	0.10	0.16	0.11	0.57	0.57
	Pills can be started immediately after birth	0.14	0.10	0.13	0.20	0.06	0.47	0.63

The results of testing the reliability of newborn and child health knowledge questions across both in-person and phone modalities are exhibited in Table 5. Questions are displayed in the order in which they appear in the questionnaire; options are ordered from highest reliability (> 0.7 for both modalities) to only reliable in-person, and then unreliable options. Efforts to compare the reliability of these modalities against initial face-to-face survey findings suggest that unreliable questions include those about number of visits to new mothers (k = .38), providing skin to skin kangaroo mother care to a baby after delivery (k = .54), reason for starting breastfeeding immediately (k = .69), and how often to breastfeed a newborn (k = .63). Reliable questions/options in-person but not over the phone include those about using a surgical blade to cut the umbilical cord (k = .71), multiple response options for complementary foods such as eggs (k = .70), and salt and sugar solution to treat diarrhea (k = .72). Reliable questions on both modalities included those about when to begin breastfeeding after delivery (k = .95), months of exclusive breastfeeding (k = .95), and when to first bathe baby (k = .60). All kappa statistics reported here are the highest values of the two modalities for each option.

**Table 5:** Kappa statistic comparisons between the face-to-face survey and test-retest newborn and child health knowledge questions.

Question	Options	Baseline Prevalence	Baseline prevalence among retest	Baseline prevalence among phone survey	Retest Prevalence	Phone Survey Prevalence	Retest Survey Adjusted Kappa	Phone Survey Adjusted Kappa
**Domain: Essential Newborn Care**								
# of visits to new mother in first week	2	0.65	0.58	0.74	0.60	0.64	0.38	0.23
# of visits to infant in first 6 weeks	6 or 7	0.82	0.65	0.88	0.73	0.88	0.49	0.65
First baby bath	= > 1 day	0.82	0.79	0.83	0.79	0.85	0.42	0.6
Baby warmth after delivery	Baby on chest	0.91	0.80	0.95	0.86	0.95	0.68	0.83
	Kangaroo care	0.41	0.26	0.55	0.27	0.60	0.54	0.21
	Dried soon after birth	0.54	0.52	0.43	0.57	0.26	0.08	0.07
Cut umbilical cord	Knife	0.02	0.01	0.01	0.01	0.03	0.95	0.91
	Surgical blade	0.13	0.09	0.12	0.09	0.14	0.71	0.59
	Scissors	0.65	0.76	0.82	0.83	0.77	0.62	0.54
	New blade	0.87	0.80	0.88	0.91	0.51	0.56	0.12
Put on cord after delivery	Mustard oil	0.03	0.05	0.01	0.05	0.02	0.79	0.94
	Other antiseptic	0.12	0.07	0.07	0.14	0.01	0.68	0.86
	Nothing	0.56	0.43	0.74	0.44	0.66	0.46	0.57
Care for preterm low birth weight baby	Frequent feeding	0.95	0.98	0.98	0.95	0.95	0.85	0.88
	13 post-natal visits	0.09	0.05	0.06	0.08	0.00	0.82	0.87
	> routine post-natal visits	0.14	0.12	0.17	0.09	0.03	0.64	0.59
	Manually express breast milk	0.14	0.12	0.12	0.14	0.03	0.55	0.73
	Skin-to-skin care	0.33	0.14	0.42	0.25	0.05	0.39	0.14
	Maintain cleanliness	0.79	0.66	0.87	0.73	0.61	0.25	0.24
	Keep the baby warm	0.43	0.37	0.57	0.46	0.50	0.13	0.11
**Domain: Infant Feeding**								
Begin breastfeeding after delivery	Immediately	0.97	0.94	0.97	0.92	1.00	0.78	0.95
Reason for starting breastfeeding	Colostrum protects & gives nutrition for child	0.94	0.87	0.95	0.87	1.00	0.69	0.90
	Mother-baby bonding	0.13	0.10	0.14	0.18	0.01	0.54	0.72
	Mothers milk protects child	0.82	0.80	0.84	0.82	0.78	0.50	0.39
	Mothers who start breastfeeding immediately recover faster	0.15	0.12	0.21	0.27	0.10	0.36	0.52
# times/day breastfeeding	Whenever baby wants	0.13	0.13	0.14	0.11	0.08	0.63	0.74
Months of exclusive breastfeeding	6	0.99	0.95	0.99	0.97	0.98	0.91	0.95
Start of complementary foods	6 months	.97	0.95	0.98	0.92	1.00	0.78	0.96
Complementary foods	Organ meat	0.01	0.00	0.01	0.01	0.00	0.98	0.99
	Other meat	0.03	0.03	0.01	0.03	0.01	0.86	0.97
	Fish	0.02	0.02	0.02	0.05	0.01	0.90	0.95
	Fortified Baby food	0.09	0.03	0.08	0.10	0.03	0.73	0.79
	Eggs	0.12	0.13	0.14	0.17	0.09	0.70	0.65
	Grain food	0.04	0.01	0.02	0.02	0.83	0.94	0.63
	Yellow/orange vegetables	0.09	0.06	0.14	0.03	0.25	0.80	0.43
	Dairy-based products	0.15	0.08	0.19	0.10	0.34	0.71	0.30
	Juice	0.28	0.14	0.23	0.23	0.09	0.38	0.41
	Root vegetables	0.22	0.20	0.30	0.26	0.48	0.32	0.07
	Clear broth	0.21	0.14	0.17	0.20	0.39	0.31	0.10
	Any other soft food	0.67	0.71	0.75	0.82	0.20	0.28	0.30
	Green vegetables	0.33	0.33	0.34	0.41	0.09	0.19	0.35
	Ripe fruit	0.17	0.22	0.26	0.32	0.10	0.19	0.38
	Not mother's milk	0.72	0.68	0.81	0.83	0.07	0.17	0.50
	Pulses or nuts	0.45	0.46	0.45	0.59	0.23	0.08	0.15
	Plain water	0.50	0.42	0.54	0.54	0.08	0.02	-0.08
	Any other fruits or vegetables	0.31	0.32	0.41	0.50	0.34	0.02	0.07
	Other liquids	0.56	0.45	0.46	0.41	0.25	0.01	0.11
**Domain: WASH/ Diarrhea**								
Diarrhea treatment	Antibiotic	0.01	0.00	0.01	0.01	0.00	0.99	0.97
	Injection	0.03	0.08	0.01	0.04	0.01	0.79	0.97
	Intravenuous (IV)	0.03	0.01	0.01	0.01	0.01	0.97	0.96
	Home remedy/ herbal medicine	0.05	0.02	0.02	0.02	0.01	0.92	0.95
	Continue breastfeeding	0.07	0.05	0.09	0.01	0.05	0.87	0.77
	Salt and sugar solution	0.19	0.08	0.19	0.12	0.02	0.72	0.61
	Give Antidiarrheals	0.21	0.24	0.32	0.11	0.01	0.41	0.34
	Other pill or syrup	0.17	0.24	0.10	0.21	0.01	0.38	0.77
	Give ORS solution	0.86	0.86	0.88	0.69	0.67	0.25	0.23
	ORS and zinc solution	0.79	0.70	0.65	0.66	0.70	0.11	0.11
3 times to wash hands	After defecation	0.96	0.91	0.98	0.95	0.98	0.73	0.92
	Before eating or feeding the child	0.94	0.91	0.95	0.89	0.98	0.63	0.86
	Before cooking or handling food	0.92	0.84	0.91	0.89	0.99	0.55	0.81

**Fig 3:**
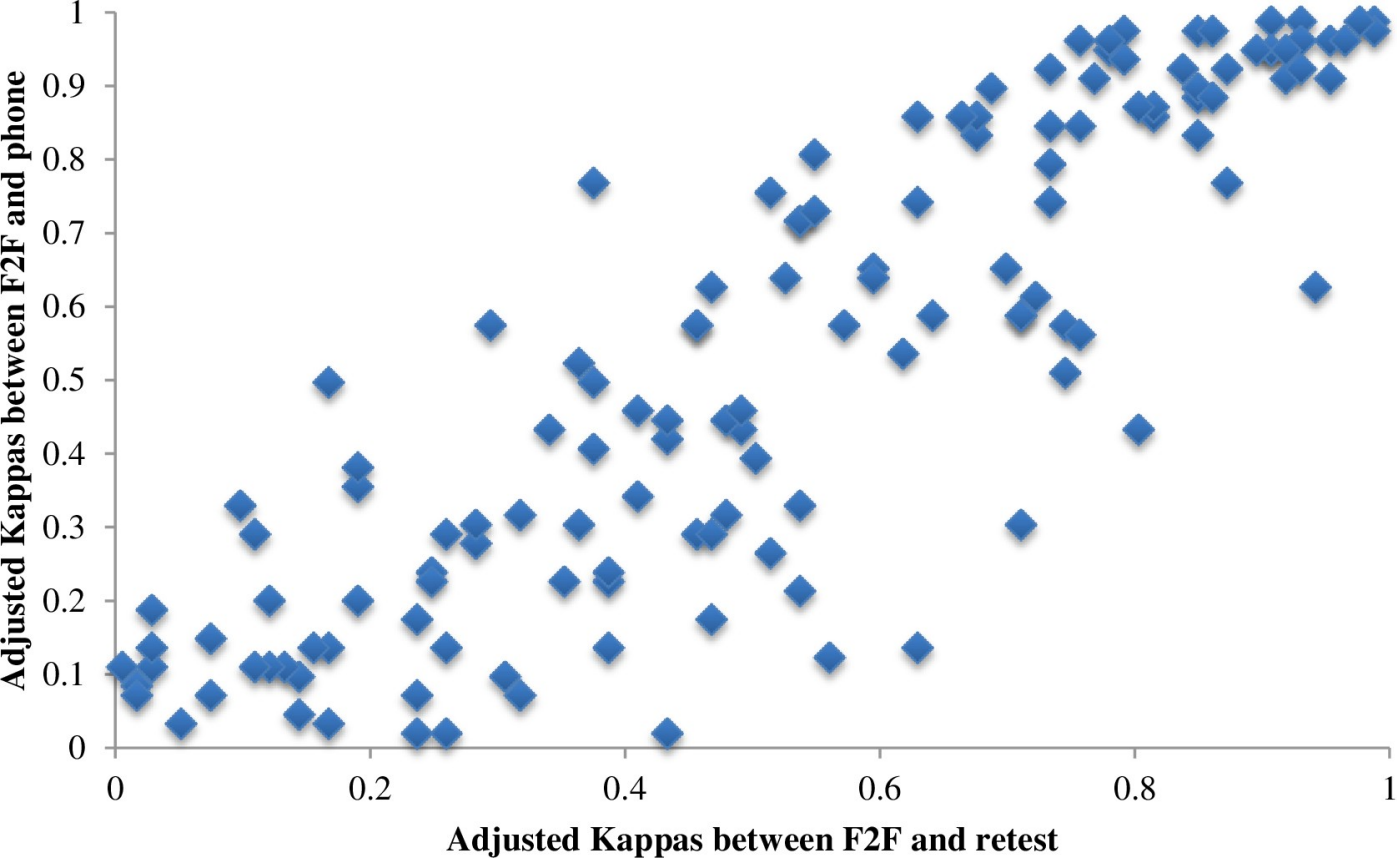
Adjusted kappa statistics comparing in-person and phone modalities for knowledge questions.

Fig 3 shows the correlation between prevalence and bias adjusted kappa statistics between (a) the test-retest and face-to-face surveys as well as (b) the phone and face-to-face surveys. Overall, the correlation between the two is high, indicating relatively few question-option pairs have very different reliabilities across modalities.

## Discussion

This study sought to examine determinants of ASHA RMNCH&N knowledge and develop a reliable phone survey tool, which could be used for the rapid, routine, and low cost measurement of knowledge. Findings from a cross-sectional survey of ASHAs in 4 districts of MP suggest RMNCH&N knowledge was highest among ASHAs closer to urban state capitol of Bhopal (in the district of Hoshangabad), who had completed 12th standard or higher education, had more experience as an ASHA, who owned a phone, could demonstrate comprehension of an IVR navigational prompt, and reported using the mobile phone to communicate for work in ways such as calling clients or messaging other ASHAs. Amongst knowledge domains assessed, ASHA average knowledge ranged between 60–71%, with the sub-domain of maternal health having the lowest average score at 60% as compared to 71% for WASH/Diarrhea or essential newborn care knowledge. The importance of knowledge impacting service delivery has been highlighted through studies on the know-do gap around tuberculosis care and childhood diarrhea and pneumonia care in India [40, 41]. Given how important knowledge is for adequate service delivery, it is necessary to measure knowledge amongst frontline health workers, who are often the bridges between communities and health systems.

Elsewhere, efforts to examine knowledge among FLHWs are emerging in the literature [12–15, 17, 19, 21–24, 42–45]. Despite a number of studies in India exploring ASHA knowledge, limitations in the domains assessed, as well as the study methods including sampling and analysis, greatly impede meaningful comparisons. In India, Kochukuttan et al. conducted a cross-sectional survey in 2011 among ASHAs (n = 225) in a rural district of Karnataka to assess knowledge of birth preparedness and pregnancy complications [17]. Amongst common indicators assessed, no knowledge of any of the key danger signs during pregnancy (Karnataka: 19%, MP: 1%) and postpartum (Karnataka: 4%, MP: 1%) was higher among ASHAs in Karnataka than in our study in MP [17]. While multivariable regressions were not carried out in the study in Karnataka to explore determinants of knowledge, bivariate analyses suggest that knowledge was significantly higher among ASHAs who had received repeated, recent and practical training [17]. A qualitative study by Ramadurg et al., also in Karnataka, highlighted gaps in knowledge on pre-eclampsia among ASHAs, staff nurses, and auxiliary nurse midwives [15]. Elsewhere in India, Kohli et al. conducted a descriptive cross-sectional study in Delhi (n = 55) on ASHAs’ knowledge of maternal health; observing higher knowledge about numbers of days to take IFA tablets as compared to MP (Delhi: 87%; MP: 20%) [42]. In Haryana, Garg et al. interviewed ASHAs about pregnancy danger signs and immunizations (n = 105) and found lower ASHA knowledge for common metrics assessed in comparison to this present study in MP, such as swelling during pregnancy being a danger sign (Haryana: 73%; MP 80%) [21]. Findings from a cross-sectional survey in Gujarat assessing knowledge of child health among ASHAs (n = 130) echo this trend, whereas ASHAs in Gujarat had slightly lower knowledge amongst common indicators of immediate breast feeding (Gujarat: 82%, MP: 97%) and complimentary feeding (Gujarat: 28%, MP: 97%) [23]. In Rajasthan, the reverse trend was observed, with findings from a cross-sectional survey on ASHA knowledge of family planning, HIV/AIDs, maternal health care, and child health care suggesting that ASHAs in Rajasthan had slightly higher knowledge than that in MP for reproductive health (Rajasthan: 91%, MP: 61%) and maternal health (Rajasthan: 87%%, MP: 60%) [24]. Outside of India, knowledge assessments have been conducted amongst primary care providers and FLHWs in Tanzania [43], Myanmar [44], and Mozambique [45]. Collectively, the evidence to date is not only reflective of varied methodological approaches to data collection and analysis, but also illuminates the wide range in knowledge of essential health topics among ASHAs in different states–a factor unsurprising given the rich diversity present within and across geographic areas in India.

To complement efforts to better understand current levels of and determinants of ASHA knowledge, we additionally sought to develop a reliable phone survey tool which could be more widely used for the routine, low cost measurement of knowledge at scale. Efforts to assess the reliability of phone survey content indicate that questions with lower reliability (<0.70 kappa statistic) included those about reasons to seek medical care for new mothers, benefits of family planning, impact of male sterilization, harmful effects of and use of IUDs, number of visits to new mothers, providing warmth to a baby after delivery, reason for starting breastfeeding immediately, how often to breastfeed a newborn, and when to start complementary feeding. Reliable questions in-person *but not* over the phone include those about number of IFA tablets taken during pregnancy, difficulty breastfeeding being a danger sign for new mothers, female sterilization and the rhythm methods as modern family planning methods, using a surgical blade to cut the umbilical cord, multiple options for complementary foods, and salt and sugar solution to treat diarrhea. Reliable questions *on both* modalities included those about number of tetanus shots during pregnancy, birth spacing, free IUDs at government facilities, when to begin breastfeeding after delivery, and months of exclusive breastfeeding.

Differences in inter-rater and inter-modal reliability may be explained in part by (a) the sensitivity of certain topical areas; (b) some content areas being difficult for enumerators to convey adequately to ASHAs; (c) challenges in recall pertaining to questions with multiple response options; and (d) length of the survey tool. In addition to these considerations, the gender of the enumerators used (males) as well as the self-reported origins of the phone enumerators (being University educated and from Delhi) may have influenced findings. Certain topical areas, including family planning, emerged as particularly challenging for enumerators to ask ASHAs about over the phone. This was attributed not only to the gender of the enumerator, but also to challenges translating family planning concepts in a way that could be understood, and as well unknowns with regard to who was in the vicinity of the ASHA at the time of the interview. While it was reported to be common for ASHAs to engage with male providers and supervisors (hence the use of male enumerators), future studies might see higher reliability via the use of female enumerators. Questions with lengthy response options, including those about maternal and infant nutrition, had lower reliability, which may be attributed to challenges recalling multiple types of food over time. We note additionally that the majority of this population is Hindu and hence mention of meat and fish were low, although reliable across modalities. Future surveys aiming to measure ASHA knowledge around nutrition might consider further breaking questions down by category to obtain more targeted and reliable responses. The length of the survey tool additionally played an important role in the reliability of results. The initial face-to-face survey spanned for just over one hour. While modules were removed to streamline the tool for test retests and phone surveys, it nevertheless spanned for 30–45 minutes. Many of the phone survey enumerators expressed difficulty encouraging ASHAs to complete the survey in one sitting given their competing service delivery and other responsibilities. Future tools should not exceed 20 minutes.

Elsewhere we have described our methods for developing the phone survey tool [46]. This approach differs from previous efforts to develop phone surveys in number of important ways [28, 47–49]. Ours is one of few studies to have explored both inter-rater and inter-modal reliability. To assess inter-rater reliability we repeated the same face-to-face survey amongst a sub-sample of respondents. This proved to be critical in determining whether questions yielded reliable responses irrespective of the modality. By further using that the same field-based cadre of enumerators for both face-to-face survey rounds, we were able to ensure that reliability measures were not confounded by differences in enumerator location (e.g. MP versus Delhi) or background characteristics. To assess inter-modal reliability, we administered the same tool over the phone to a sub-sample of ASHAs interviewed during the initial face-to-face survey. Elsewhere, efforts to develop phone survey tools have assessed intermodal reliability amongst the same sample. In Lebanon, Mahfoud et al. compared the reliability of face-to-face versus phone surveys for self-reported chronic conditions. Results suggest that questions about age, health insurance, education, and cigarette smoking had high reliability (kappa statistic > 0.80) across both modalities using independent samples of n = 630 each [47]. While they did not include a step assessing inter-rater reliability (test retest), they nevertheless developed an abridged version of the face-to-face interview, and ultimately, demonstrated the feasibility and reliability of using cellphones to connect with people who have previously been interviewed face-to-face. In Brazil, Francisco et al. found that self-reported chronic conditions were reported at the same or higher prevalence via phone modality as compared to face-to-face interviews and were compared using independent t-tests [48]. This study did not use the kappa statistics, and did not assess reliability among the same respondents.

To our knowledge, this is the first study of its kind aiming to develop a reliable phone survey tool for the routine assessment of knowledge amongst FLHWs in India. A 2017 systematic review by Greenleaf et al. sought to compare the reliability of in-person and remote survey modalities; however, no articles were found specific to the measurement of FLHW knowledge using phone surveys. Du et al. assessed reliability using the kappa statistic between a face-to-face survey and test messages in China of knowledge about young infant feeding among mothers [50]. Another study based in China explored reliability of questions posed to participants of an infant feeding health education program rather than focusing on FLHWs’ knowledge [51]. Other articles mentioned in the review focused on assessing disease prevalence using these alternate modalities without adequate emphasis on the content of the tool itself [49]. Efforts to measure intermodal reliability were reported in 5 out of the 10 articles which used face-to-face surveys and then CATI surveys, of which 3 were independent samples and 2 very dependent samples [49]. While we did not do a cost assessment, a mobile phone survey with community health workers in Malawi found that cost per interview of the mobile phone methodology is considerably less than what it would cost to conduct in-person [52]. Given the largeness and high mobile connectivity of India, cost reductions in are especially helpful. The lack of CATI surveys assessing frontline health worker knowledge in the literature indicates the need for this type of research. More broadly, our findings are a promising addition to the scant literature indicating that phone surveys may provide a reliable alternative to more costly, time intensive face-to-face modalities. However, careful attention to the content must be paid to ensure data quality.

### Limitations and future research

There are several notable limitations in our study. Our approach to measuring knowledge and determinants of knowledge was limited to RMNCH&N domains, and stopped short of exploring ASHA practices, examining interpersonal communication, and studying the translation of knowledge and practices to beneficiaries. We assumed ASHAs responded to knowledge questions with government recommendations in mind, rather than voicing personal opinions; however, further cognitive testing could have examined if this were truly the case. Because of resource limitations, cognitive testing–whilst done for other phone surveys developed as part of this project–was not feasible to undertake here [53]. Cognitive testing enables researchers to evaluate whether quantitative survey questions are generating the information that the researchers intend by exploring whether the questions are accessing the same cognitive domains among respondents as expected by the survey developers [54]. Amongst determinants assessed, ASHAs were asked about exposure to Mobile Academy–a mobile health training initiative carried out across 13 states–but stopped short of inquiring about other forms of capacity building and training received due to survey length constraints. While linkages between Mobile Academy and knowledge were not directly seen, this is likely attributed to the rollout of Mobile Academy as having occurred in 2016 and resulting in lower penetration in MP as compared to other states. Exposure to training overall is anticipated to be highly associated with knowledge and future surveys should explore exposure to multiple forms of training as an important determinant of knowledge.

Data collection was restricted to four districts of MP, selected as part of a larger impact evaluation [32], which may have implications for the generalizability of findings. Additionally, our phone survey came after an initial interview in person; response rates and comfort with sensitive topics may not have been as high had we not first interviewed the ASHA in person. Efforts to develop reliable phone survey focused on inter-rater and inter-modal reliability of face-to-face versus CATI surveys. Lower cost alternatives such as Short Messaging Service (SMS) surveys were not assessed given concerns about literacy and digital literacy of ASHAs. Male enumerators served as the point of contact for all surveys–a factor which may have influenced ASHA responses. Phone surveys were carried out from Delhi by students from a university in Delhi who have a different language and socio-demographic profiles than respondents–factors which too may have influenced emerging findings as noted above. Future surveys could consider all female enumerators to reduce any potential awkwardness around sensitive subjects. Call drop rates were low but were not formally examined; a deeper understanding of call drop rate and reason could maximize responses in future studies. We used a binary threshold for examining reliability above or below .7; however, reliability can also be examined at a more granular level using multiple classifications of reliability as described by Watson et al. [55]. Further examination of low reliability questions is required to identify the underlying reasons for different responses and to develop more reliable alternatives. In case of questions with a high number of response options, ASHA may not spontaneously recall all the answers. Questions containing difficult concepts to convey by enumerators may benefit from cognitive testing to further nuance and improve the wording and translation used.

## Conclusion

Understanding the determinants of knowledge among ASHAs is important for identifying critical gaps which may impede service delivery and in-turn the quality of RMNCH&N services provided in India. Overall results suggest that ASHAs may benefit from additional training across a number of areas, including maternal health and family planning, both key areas of the continuum of care to improve health among maternal and neonatal health. Recent policies to increase the scope of work of ASHAs as part of Home Based Care for Young Child [56] as well as the new Comprehensive Primary Health Care through Health and Wellness Centers may provide an opportunity for such training. Efforts to develop a reliable phone survey tool for measuring RMNCH&N knowledge suggest that reliability was higher for shorter, basic questions, including those about timing of exclusive breastfeeding or number of tetanus shots during pregnancy rather than longer questions about harmful effects of intrauterine devices. Overall results highlight important gaps in the knowledge of ASHAs. This research has led to the development of a tool, which can now be widely used for the routine, low-cost measurement of ASHA RMNCH&N knowledge in India, identifying important topical targets for focused in-service refresher trainings. As phone-based assessments may be relatively cheaper and faster to deploy, we can imagine a future where bespoke phone-based training is offered to ASHAs based on individual knowledge gaps, improving the overall capacity of this important frontline cadre in India.

## Supporting information

S1 File Codebook for measuring ASHA knowledge data. (DOCX)

S1 Table (CSV)
