# Supplementary material for: Novel approaches to measuring knowledge among frontline health workers in India: Are phone surveys a reliable option?
Source: PLoS One. 2020 Jun 29;15(6):e0234241. doi: 10.1371/journal.pone.0234241 (PMC7323989; doi:10.1371/journal.pone.0234241)
Supplement: S1 File — (DOCX) [file pone.0234241.s001.docx]

Codebook for Measuring ASHA Knowledge Data

district_as: variable signifying one of four districts including Mandsaur, Rajgarh, Hoshangabad, and Rewa

distBorn: Born in the same district as which they work as an ASHA in signified by yes, otherwise 0 signifies no

numChild: An ASHAs parity or number of current children as a continuous variable

ownPhone: Indicates if a ASHA owns her phone (Yes) or is she does not (0)

govSIM: Indicates if an ASHA has a government SIM (Yes) or not (0)

govPhone: Indicates if an ASHA has a government phone (Yes) or not (0)

HHwealthindex: Quintiles of wealth in the sample with 1 being the poorest and 5 being the richest.

Caste: The caste of the ASHA as split into three categories: general caste, scheduled caste, other backwards class

matHealthScore: The created score of maternal health knowledge out of 100 based on her answers to 7 questions in the knowledge module

encp: The created percent score of essential newborn care knowledge out of 100% based on her answers to 7 questions

feedingScorep: The created score of maternal health knowledge out of 100 based on her answers to 6 questions in the knowledge module

fpScorep: The created score of maternal health knowledge out of 100 based on her answers to 12 questions in the knowledge module

washScorep: The created score of maternal health knowledge out of 100 based on her answers to 2 questions in the knowledge module

totalScorep: The created score of maternal health knowledge out of 100 based on her answers to 34 questions in the knowledge module

married: 1 if married or in a marital union; 0 if otherwise

edu4: Education as split up into 4 categories – primary or less, middle school, high school, higher education

asha_exp: Respondents duration as an ASHA worker split up into 3 categories—0-4 years, 5-10 years, or 11+ years.

age1: Age up till 30 years

age2: Age beyond 30 years

lit: 1 – can read a full sentence; 0 – cannot read a full sentence

weekVisits: If an ASHA visits her clients 7 or more times a week then 1, otherwise 0

MA: Has heard of Mobile Academy – 1; otherwise 0

iMA: Has started Mobile Academy training program -1 ; otherwise 0

completeMA: ASHA has completed Mobile Academy training program – ; otherwise 0

smartphone: ASHA owns a smartphone – 1; otherwise 0

dailyPhone: She uses a phone daily – 1; otherwise 0

dialMiss: ASHA was able to demonstrate the ability to dial a missed call – 1; otherwise 0

understoodMsg: ASHA was able to demonstrate the ability to understand a navigational prompt on her phone – 1; otherwise 0

openSMS: ASHA was able to demonstrate the ability to open a SMS message on her phone

readSMS: ASHA was able to demonstrate the ability to read an SMS message on her phone

SMS: ASHA was able to demonstrate the ability to both read and open an SMS

storeContacts: ASHA was able to demonstrate the ability to store contacts on her phone

workPhone: A score out of ten of the ways in which ASHAs use their phones for work including calling pregnant or lactating women, communicating with ANM/ASHA Facilitator, communicating with Anganwadi Worker/ Other ASHAs, communicating with Block-level officials, communicating with other health workers, SMS with pregnant or lactating women, SMS with ANM/ASHA Facilitator, SMS with Anganwadi Worker/ Other ASHAs, SMS with Communicate with Block-level official, or SMS with health workers.

Knowledge section: These variables will either have a nothing before them to indicate the baseline knowledge, ‘p_’ to indicate knowledge collected through the phone surveys, or ‘r_’ to indicate knowledge collected through the test-retest survey.

q301_dmy_A: Indicates that ASHA thinks pregnancy women should eat Milk/Dairy products - 1: 0 otherwise

q301_dmy_B: Indicates that ASHA thinks pregnancy women should eat Green leafy vegetables - 1: 0 otherwise

q301_dmy_C: Indicates that ASHA thinks pregnancy women should eat Fruits - 1: 0 otherwise

q301_dmy_D: Indicates that ASHA thinks pregnancy women should eat Pluses and nuts - 1: 0 otherwise

q301_dmy_E: Indicates that ASHA thinks pregnancy women should eat Non-vegetarians: eggs - 1: 0 otherwise

q301_dmy_F: Indicates that ASHA thinks pregnancy women should eat Non-vegetarians: meats - 1: 0 otherwise

q301_dmy_G: Indicates that ASHA thinks pregnancy women should eat Non-vegetarians: fish - 1: 0 otherwise

q302: Number of tetanus injections a women should get during pregnancy ; where 1 denotes the correct answer of 2 and 0 indicates the wring answer was selected

correctIFAno: Number of iron folic acid tablets a women should take during pregnancy

q304_dmy_A: Indicates that ASHA thinks pregnant women should take Iron Folic Acid tablets during pregnancy to help prevent/treat anemia – 1; 0 is they did not mention this option.

q304_dmy_B: Indicates that ASHA thinks pregnant women should take Iron Folic Acid tablets during pregnancy to help improve the health / well-being of my baby – 1; 0 is they did not mention this option.

q305_dmy_A: Indicates that ASHA thinks pregnant women and her family should prepare for the delivery by registering the pregnancy – 1; 0 is they did not mention this option.

q305_dmy_B: Indicates that ASHA thinks pregnant women and her family should prepare for the delivery by identifying transportation to health clinic – 1; 0 is they did not mention this option.

q305_dmy_C: Indicates that ASHA thinks pregnant women and her family should prepare for the delivery by identifying place of delivery in advance – 1; 0 is they did not mention this option.

q305_dmy_D: Indicates that ASHA thinks pregnant women and her family should prepare for the delivery by keeping ASHA’s number – 1; 0 is they did not mention this option.

q305_dmy_E: Indicates that ASHA thinks pregnant women and her family should prepare for the delivery by saving money for emergency – 1; 0 is they did not mention this option.

q305_dmy_F: Indicates that ASHA thinks pregnant women and her family should prepare for the delivery by keeping clothes ready – 1; 0 is they did not mention this option.

q305_dmy_G: Indicates that ASHA thinks pregnant women and her family should prepare for the delivery by identifying family members to accompany you to facility – 1; 0 is they did not mention this option.

q306_dmy_A: Indicates that ASHA know vaginal bleeding is one of the danger signs **during pregnancy** for which pregnant women should go to a health facility immediately – 1; 0 is they did not mention this option.

q306_dmy_B: Indicates that ASHA know vaginal discharge is one of the danger signs **during pregnancy** for which pregnant women should go to a health facility immediately – 1; 0 is they did not mention this option.

q306_dmy_C: Indicates that ASHA know convulsions is one of the danger signs **during pregnancy** for which pregnant women should go to a health facility immediately – 1; 0 is they did not mention this option.

q306_dmy_D: Indicates that ASHA know stomach cramps are one of the danger signs **during pregnancy** for which pregnant women should go to a health facility immediately – 1; 0 is they did not mention this option.

q306_dmy_E: Indicates that ASHA know swelling on limbs and face is one of the danger signs **during pregnancy** for which pregnant women should go to a health facility immediately – 1; 0 is they did not mention this option.

q306_dmy_F: Indicates that ASHA know yellowing of skin is one of the danger signs **during pregnancy** for which pregnant women should go to a health facility immediately – 1; 0 is they did not mention this option.

q306_dmy_G: Indicates that ASHA know fever is one of the danger signs **during pregnancy** for which pregnant women should go to a health facility immediately – 1; 0 is they did not mention this option.

q306_dmy_H: Indicates that ASHA know decreased or absent fetal movements is one of the danger signs **during pregnancy** for which pregnant women should go to a health facility immediately – 1; 0 is they did not mention this option.

q306_dmy_I: Indicates that ASHA know dizziness and headache are one of the danger signs **during pregnancy** for which pregnant women should go to a health facility immediately – 1; 0 is they did not mention this option.

correct_q307: ASHAs who said that newborns should be visited two times in the first week after delivery – 1; 0 if mention any other number of times.

correct_q308: ASHAs who said that newborns should be visited six or seven times within the first six week after delivery – 1; 0 if mention any other number of times.

q309: Knowledge of when to start breastfeeding after birth—where immediately is correct and 0 is an incorrect response

q310_dmy_A: ASHAs who know that a reason for starting early breastfeeding is that the colostrum protects the child and is good nutrition for the child – 1; 0 if they do not give this option.

q310_dmy_B: ASHAs who know that a reason for starting early breastfeeding is that the Mothers milk can protect the child – 1; 0 if they do not give this option.

q310_dmy_C: ASHAs who know that a reason for starting early breastfeeding is that is encourages Mother-baby bonding – 1; 0 if they do not give this option.

q310_dmy_D: ASHAs who know that a reason for starting early breastfeeding is Mothers who start breastfeeding immediately recover faster after childbirth – 1; 0 if they do not give this option.

q311: Knowledge of how many times per day should newborn babies be breastfed, where 9-12 times is the correct response, 0 indicates an incorrect response was chosen.

Q312: 1 indicates knowledge of 6 months of exclusive breastfeeding and 0 indicates lack of knowledge

Correct_Q313: Newborns who were bathed more than a day after delivery – 1; 0 if ASHAs did not provide this answer.

Q314_dmy_A: ASHAs who know that a method to keep a baby warm immediately after birth is to ensure they are dried or wiped soon after birth – 1; 0 if ASHAs did not provide this answer.

Q314_dmy_B: ASHAs who know that a method to keep a baby warm immediately after birth is to ensure they put the baby on a mother’s chest – 1; 0 if ASHAs did not provide this answer.

Q314_dmy_C: ASHAs who know that a method to keep a baby warm immediately after birth is to ensure they use Kangaroo care – 1; 0 if ASHAs did not provide this answer.

Q316_dmy_A: ASHAs who know that new blade can be used to cut an umbilical cord – 1; 0 if ASHAs did not provide this answer.

Q316_dmy_B: ASHAs who said that a blade used for other purposes can be used to cut an umbilical cord – 1; 0 if ASHAs did not provide this answer.

Q316_dmy_C: ASHAs who said that scissors can be used to cut an umbilical cord – 1; 0 if ASHAs did not provide this answer.

Q316_dmy_D: ASHAs who said that a knife can be used to cut an umbilical cord – 1; 0 if ASHAs did not provide this answer.

Q316_dmy_E: ASHAs who said that a surgical blade can be used to cut an umbilical cord – 1; 0 if ASHAs did not provide this answer.

Q317_dmy_A: ASHAs who said that chlorohexidine should be put on the cord after delivery – 1; 0 if ASHAs did not provide this answer.

Q317_dmy_B: ASHAs who said that other antiseptic (alcohol, spirit, gentian violet) should be put on the cord after delivery – 1; 0 if ASHAs did not provide this answer.

Q317_dmy_C: ASHAs who said that other mustard oil should be put on the cord after delivery – 1; 0 if ASHAs did not provide this answer.

Q317_dmy_D: ASHAs who said that other ash should be put on the cord after delivery – 1; 0 if ASHAs did not provide this answer.

Q317_dmy_E: ASHAs who said that other dung should be put on the cord after delivery – 1; 0 if ASHAs did not provide this answer.

Q317_dmy_F: ASHAs who said that nothing should be put on the cord after delivery – 1; 0 if ASHAs did not provide this answer.

Q318_dmy_A: ASHA who said that frequent feeding is an important step mothers must take to care for a preterm/low birth weight baby – 1; 0 if ASHA did not provide this answer.

Q318_dmy_B: ASHA who said that skin-to-skin care for several hours is an important step mothers must take to care for a preterm/low birth weight baby – 1; 0 if ASHA did not provide this answer.

Q318_dmy_C: ASHA who said 13 post-natal visits is an important step mothers must take to care for a preterm/low birth weight baby – 1; 0 if ASHA did not provide this answer.

Q318_dmy_D: ASHA who said more than routine post-natal visits is an important step mothers must take to care for a preterm/low birth weight baby – 1; 0 if ASHA did not provide this answer.

Q318_dmy_E: ASHA who said manually expressing breast milk and feed the baby if the baby is too weak to suckle is an important step mothers must take to care for a preterm/low birth weight baby – 1; 0 if ASHA did not provide this answer.

Q318_dmy_F: ASHA who said maintain cleanliness is an important step mothers must take to care for a preterm/low birth weight baby – 1; 0 if ASHA did not provide this answer.

Q318_dmy_G: ASHA who said keeping the baby warm is an important step mothers must take to care for a preterm/low birth weight baby – 1; 0 if ASHA did not provide this answer.

Q319_dmy_A: ASHA who said that fever is one of the signs for which a newly delivered mother should seek care or go to a health facility - 1; 0 if ASHAs did not provide this answer.

Q319_dmy_B: ASHA who said that breathlessness/difficulty breathing/fatigue is one of the signs for which a newly delivered mother should seek care or go to a health facility - 1; 0 if ASHAs did not provide this answer.

Q319_dmy_C: ASHA who said that Abnormal behavior (depression, psychosis) is one of the signs for which a newly delivered mother should seek care or go to a health facility - 1; 0 if ASHAs did not provide this answer.

Q319_dmy_D: ASHA who said that Placenta not delivered within one hour after delivery is one of the signs for which a newly delivered mother should seek care or go to a health facility - 1; 0 if ASHAs did not provide this answer.

Q319_dmy_E: ASHA who said that Painful breast/bleeding nipple/ difficulty breastfeeding is one of the signs for which a newly delivered mother should seek care or go to a health facility - 1; 0 if ASHAs did not provide this answer.

Q319_dmy_F: ASHA who said that Foul smelling vaginal discharge is one of the signs for which a newly delivered mother should seek care or go to a health facility - 1; 0 if ASHAs did not provide this answer.

Q319_dmy_G: ASHA who said that Headache/blurred vision is one of the signs for which a newly delivered mother should seek care or go to a health facility - 1; 0 if ASHAs did not provide this answer.

Q319_dmy_H: ASHA who said that Loss of consciousness is one of the signs for which a newly delivered mother should seek care or go to a health facility - 1; 0 if ASHAs did not provide this answer.

Q319_dmy_I: ASHA who said that Convulsions/fits is one of the signs for which a newly delivered mother should seek care or go to a health facility - 1; 0 if ASHAs did not provide this answer.

Q319_dmy_J: ASHA who said that Severe pain in abdomen/genital region is one of the signs for which a newly delivered mother should seek care or go to a health facility - 1; 0 if ASHAs did not provide this answer.

Q319_dmy_K: ASHA who said that Scanty urine (oliguria) is one of the signs for which a newly delivered mother should seek care or go to a health facility - 1; 0 if ASHAs did not provide this answer.

Q319_dmy_L: ASHA who said that Excessive vaginal bleeding

is one of the signs for which a newly delivered mother should seek care or go to a health facility - 1; 0 if ASHAs did not provide this answer.

correct_q320: Said that on average new mothers should wait at least 3 years before having another child – 1; 0 if said anything else.

Q321_dmy_A: ASHA mentioned that one of the benefits of family planning is that it is an easy way to control the size of your family - 1; 0 if ASHAs did not provide this answer.

Q321_dmy_B: ASHA mentioned that one of the benefits of family planning is that it can give you more time to take care of the children you already have - 1; 0 if ASHAs did not provide this answer.

Q321_dmy_C: ASHA mentioned that one of the benefits of family planning is that it can provide financial savings - 1; 0 if ASHAs did not provide this answer.

Q322_dmy_A: ASHA mentioned female sterilization as one of the modern family planning methods she knows of - 1; 0 if ASHAs did not provide this answer.

Q322_dmy_B: ASHA mentioned male sterilization as one of the modern family planning methods she knows of - 1; 0 if ASHAs did not provide this answer.

Q322_dmy_C: ASHA mentioned IUCD/Copper T/PPIUCD as one of the modern family planning methods she knows of - 1; 0 if ASHAs did not provide this answer.

Q322_dmy_D: ASHA mentioned Oral contraceptive pills as one of the modern family planning methods she knows of - 1; 0 if ASHAs did not provide this answer.

Q322_dmy_E: ASHA mentioned Injectables as one of the modern family planning methods she knows of - 1; 0 if ASHAs did not provide this answer.

Q322_dmy_F: ASHA mentioned Condom/Nirodh as one of the modern family planning methods she knows of - 1; 0 if ASHAs did not provide this answer.

Q322_dmy_G: ASHA mentioned Rhythm method as one of the modern family planning methods she knows of - 1; 0 if ASHAs did not provide this answer.

Q322_dmy_H: ASHA mentioned Withdrawal as one of the modern family planning methods she knows of - 1; 0 if ASHAs did not provide this answer.

Q322_dmy_I: ASHA mentioned Lactational amenorrhea method as one of the modern family planning methods she knows of - 1; 0 if ASHAs did not provide this answer.

Q323_dmy_A: ASHA mentioned that it is not possible to use family planning methods immediately after birth -1; 0 if ASHAs did not provide this answer.

Q323_dmy_B: ASHA mentioned that Female sterilization is a possible family planning method that is available right after delivier to a new mother before leaving the facility -1; 0 if ASHAs did not provide this answer.

Q323_dmy_C: ASHA mentioned that male sterilization is a possible family planning method that is available right after delivier to a new mother before leaving the facility -1; 0 if ASHAs did not provide this answer.

Q323_dmy_D: ASHA mentioned that IUD is a possible family planning method that is available right after delivier to a new mother before leaving the facility -1; 0 if ASHAs did not provide this answer.

Q323_dmy_E: ASHA mentioned that oral contraceptive pills are a possible family planning method that is available right after delivier to a new mother before leaving the facility – 1; 0 if ASHAs did not provide this answer.

Q323_dmy_F: ASHA mentioned that injectables are a possible family planning method that is available right after delivery to a new mother before leaving the facility -1; 0 if ASHAs did not provide this answer.

Q323_dmy_G: ASHA mentioned that condom/nirodh is a possible family planning method that is available right after delivery to a new mother before leaving the facility – 1; 0 if ASHAs did not provide this answer.

Q323_dmy_H: ASHA mentioned that rhythm method is a possible family planning method that is available right after delivery to a new mother before leaving the facility -1; 0 if ASHAs did not provide this answer.

Q323_dmy_I: ASHA mentioned that withdrawal is a possible family planning method that is available right after delivery to a new mother before leaving the facility – 1; 0 if ASHAs did not provide this answer.

Q323_dmy_J: ASHA mentioned that Lactational amenorrhoea method is a possible family planning method that is available right after delivery to a new mother before leaving the facility – 1; 0 if ASHAs did not provide this answer.

Q324a: True: ASHAs who think male sterilization is an easy way to control family size; 0 – ASHAs think this is false

Q324b: True: Men become physically weak after accepting male sterilization; 0 – ASHAs think this is false

Q324c: True: PPIUCD insertion and female sterilization services can be free of cost at government facilities; 0 – ASHAs think this is false

Q324d: True: PPIUCD has **many harmful** side effects; 0 – ASHAs think this is false

Q324e: True: Women may never be able to have another baby if they use IUD for family planning; 0 – ASHAs think this is false

Q324f: True: IUDs can be used for up to 10 years, if needed; 0 – ASHAs think this is false

Q325_dmy_A: ASHA reported that when she is sharing information with women about injectable contraceptives (Antara/Dimpa), she mentions They are safe to use for breastfeeding mothers -1, 0 if ASHAs did not provide this answer.

Q325_dmy_B: ASHA reported that when she is sharing information with women about injectable contraceptives (Antara/Dimpa), she mentions they xan be started 6 weeks after childbirth -1, 0 if ASHAs did not provide this answer.

Q325_dmy_C: ASHA reported that when she is sharing information with women about injectable contraceptives (Antara/Dimpa), she mentions they provides protection from pregnancy for 3 months -1, 0 if ASHAs did not provide this answer.

Q325_dmy_D: ASHA reported that when she is sharing information with women about injectable contraceptives (Antara/Dimpa), she mentions they should be taken every 3 month -1, 0 if ASHAs did not provide this answer.

Q326_dmy_A: ASHA reported that when she is sharing information with women about oral contraceptive pills (Mala-N), she mentions Pills can be started immediately after birth -1, 0 if ASHAs did not provide this answer.

Q326_dmy_B: ASHA reported that when she is sharing information with women about oral contraceptive pills (Mala-N), she mentions these are safe options for breastfeeding mothers -1, 0 if ASHAs did not provide this answer.

Q326_dmy_C: ASHA reported that when she is sharing information with women about oral contraceptive pills (Mala-N), she mentions Mala-N/OCP should be taken with regularity as prescribed -1, 0 if ASHAs did not provide this answer.

Q327_dmy_A: ASHA said that ORS solution should be given to a child to treat diarrhea - 1; 0 if ASHAs did not provide this answer.

Q327_dmy_B: ASHA said that ORS and zinc solution should be given to a child to treat diarrhea - 1; 0 if ASHAs did not provide this answer.

Q327_dmy_C: ASHA said that Salt and sugar solution should be given to a child to treat diarrhea - 1; 0 if ASHAs did not provide this answer.

Q327_dmy_D: ASHA said that Antidiarrheals should be given to a child to treat diarrhea - 1; 0 if ASHAs did not provide this answer.

Q327_dmy_E: ASHA said that Antibiotics should be given to a child to treat diarrhea - 1; 0 if ASHAs did not provide this answer.

Q327_dmy_F: ASHA said that Other pill or syrup should be given to a child to treat diarrhea - 1; 0 if ASHAs did not provide this answer.

Q327_dmy_G: ASHA said that Injection should be given to a child to treat diarrhea - 1; 0 if ASHAs did not provide this answer.

Q327_dmy_H: ASHA said that Intravenuous (IV) should be given to a child to treat diarrhea - 1; 0 if ASHAs did not provide this answer.

Q327_dmy_I: ASHA said that Home remedy/ herbal medicine should be given to a child to treat diarrhea - 1; 0 if ASHAs did not provide this answer.

Q327_dmy_J: ASHA said that Continue breastfeeding should be given to a child to treat diarrhea - 1; 0 if ASHAs did not provide this answer.

Q328: ASHAs that know baby be given foods other than mother’s milk for the first time at 6 months; 0 if gave incorrect time

Q329_dmy_A: ASHA said that plain water should be given to a baby over six months – 1; 0 if did not give this option.

Q329_dmy_B: ASHA said that Juice or juice drinks should be given to a baby over six months – 1; 0 if did not give this option.

Q329_dmy_C: ASHA said that Clear broth should be given to a baby over six months – 1; 0 if did not give this option.

Q329_dmy_D: ASHA said that Milk such as tinned, powdered, or fresh animal milk should be given to a baby over six months – 1; 0 if did not give this option.

Q329_dmy_E: ASHA said that Other liquids should be given to a baby over six months – 1; 0 if did not give this option.

Q329_dmy_F: ASHA said that Any commercially fortified baby food, e.g. Cerelac or Farex should be given to a baby over six months – 1; 0 if did not give this option.

Q329_dmy_G: ASHA said that Any bread, roti, chapati, rice, noodles, biscuits, idli, or any other foods made from grains should be given to a baby over six months – 1; 0 if did not give this option.

Q329_dmy_H: ASHA said that Any pumpkin, carrots, squash or sweet potatoes that are yellow or orange inside should be given to a baby over six months – 1; 0 if did not give this option.

Q329_dmy_I: ASHA said that Any white potatoes, white yams, manioc, cassava, or any other foods made from roots should be given to a baby over six months – 1; 0 if did not give this option.

Q329_dmy_J: ASHA said that Any dark green, leafy vegetables should be given to a baby over six months – 1; 0 if did not give this option.

Q329_dmy_K: ASHA said that Any ripe mangoes, papayas, cantaloupe or jackfruit should be given to a baby over six months – 1; 0 if did not give this option.

Q329_dmy_L: ASHA said that Any other fruits or vegetables should be given to a baby over six months – 1; 0 if did not give this option.

Q329_dmy_M: ASHA said that Any liver, kidney, heart or other organ meat should be given to a baby over six months – 1; 0 if did not give this option.

Q329_dmy_N: ASHA said that Any chickens, duck, or other birds should be given to a baby over six months – 1; 0 if did not give this option.

Q329_dmy_O: ASHA said that Any other meat should be given to a baby over six months – 1; 0 if did not give this option.

Q329_dmy_P: ASHA said that Any eggs should be given to a baby over six months – 1; 0 if did not give this option.

Q329_dmy_Q: ASHA said that Any fresh or dried fish or shellfish should be given to a baby over six months – 1; 0 if did not give this option.

Q329_dmy_R: ASHA said that Any foods made from beans, peas, lentils, or nuts should be given to a baby over six months – 1; 0 if did not give this option.

Q329_dmy_S: ASHA said that Any cheese or other food made from milk should be given to a baby over six months – 1; 0 if did not give this option.

Q329_dmy_T: ASHA said that Any other solid, semi-solid, or soft food should be given to a baby over six months – 1; 0 if did not give this option.

Q330_dmy_A: ASHA said that After defecation is a critical time for a woman to wash her hands - 1; 0 – did not give this option.

Q330_dmy_B: ASHA said that Before cooking or handling food is a critical time for a woman to wash her hands - 1; 0 – did not give this option.

Q330_dmy_C: ASHA said that Before eating or feeding the child is a critical time for a woman to wash her hands - 1; 0 – did not give this option.

phonesurvey: A variable indicating if the ASHA was interviewed in the phone survey – 1; 0 if not interviewed in the phone survey.

retestsurvey: A variable indicating if the ASHA was interviewed in the test-retest survey – 1; 0 if not interviewed in the phone survey.
